# Evaluation of ERA5 wind parameter with in-situ data offshore China

**DOI:** 10.1371/journal.pone.0317751

**Published:** 2025-05-09

**Authors:** Weidong Ji, Rongfu Li, Wenfei Xue, Zhigang Cao, Hongying Yang, Qiaozhen Ning, Xiaokai Hu, Guanghong Liao

**Affiliations:** 1 Goldwind Science & Technology Co., Ltd, Urumqi, China; 2 NARI Technology Co. Ltd, Nanjing, China; 3 Key Laboratory of Marine Hazards Forecasting, Ministry of Natural Resources, Hohai University, Nanjing, China; Arab Academy for Science Technology and Maritime Transport, EGYPT

## Abstract

There are abundant wind energy resources along the coast of China. Understanding spatial-temporal characteristics of wind speed is significant in meteorology, coastal engineering design and maritime industries. Reliable wind products such as reanalysis data, coupled with accurate wind speed measurements, are essential for elucidating the primary characteristics of the wind field. In this study, we evaluated hourly 10 m and 100 m wind speed data from the fifth-generation ECMWF atmospheric reanalysis (ERA5) by comparing it with direct wind measurements obtained from 19 wind tower located across the coastal waters of China. The results are as follows: 1) the basic statistical characteristic between ERA5 reanalysis and observed wind speeds demonstrate good consistency. However, the ERA5 tends to underestimate wind speed, particularly at high speeds during extreme conditions. 2) Compare ERA5 data with observations from each station using a frequency distribution-based score method, hourly scores of most stations are between 0.8 to 0.9. It shows the higher simulation skill in the northern region than the southern due to the influence of high-frequency typhoon in the South China Sea. 3)Distribution function parameters, mean values, variability, and wind threshold frequencies were analyzed for this ensemble of observation, providing an overall description of wind characteristics. Generally speaking, there is no clear linear relationship between scores and the other variables. On longer time scales (6–24 hours), the score and correlation between ERA5 and observations further increased, while the centered root-mean-square error (CRMSE) and standard deviation decrease. 4) Hourly wind data with a regular spatial distribution in ERA5 reanalysis provides valuable information for further detailed research on meteorology or renewable energy perspectives, but some inherent shortcomings should be considered.

## 1. Introduction

Winds are a significant component of the climate system and are closely intertwined with our daily lives. They vary across a wide range of spatial and temporal scales, from the global scale to regional winds, and down to locally smaller turbulent eddies. Winds drive the ocean and are a significant source of oceanic energy [1]. Winds are also crucial components of the climate system, regulating energy the flux between the air-sea interface [2] and affecting upper ocean mixing [3]. Consequently, they affect the mechanisms of air-sea interaction [4] and have a substantial impact on global and regional climate systems. Extreme winds, such as those in Typhoons, pose a significant threat to human life and livelihood, causing extensive damage. Understanding wind and induced wave and current fields is crucial for coastal engineering design and maritime industry. Therefore, a deep insight into spatial-temporal characteristic and related aspects of wind dynamics is of great significance for multiple fields, including climate science, meteorology, oceanography sciences, and engineering [5–8].

Direct measurements of wind are rare. Previous wind field studies have relied on various techniques to obtain data, including in-situ measurements using buoys, voluntary ship observations, satellite observation, and numerical simulations. Some studies have analyzed wind speed variability in China based on different observational data and method [9–12]. However, the focus on coastal water in China is quite limited, as observations are difficult and scarce. The wind in China's coastal areas is characterized by a seasonal cycle, featuring typical southwest monsoon in summer and northeast monsoon in winter [13]. Generally, the annual averaged maximum wind speed occurs in winter [10,12].

Reanalysis data generated using data assimilation techniques that combine numerical models with observation data is an important source for wind variation studies [14]. Reanalysis products and climate models generate physically coherent atmospheric fields, particularly wind, in a continuous domain, and are characterized by long time series, high spatial and temporal resolution, and wide coverage [14]. There are several reanalysis data produces, including the Fifth Generation Global Atmospheric reanalysis data (ERA5) developed by the European Center for Medium-Range Weather Forecasting (ECMWF), the Japanese 55-year Reanalysis (JRA55), Climate Forecast System Reanalysis (CFSR) from National Centers for Environmental Prediction (NCEP), etc [15–17]. Reanalysis products are currently widely used as a good choice for providing a gridded surface wind dataset and various other atmospheric variables, to supplement the existing wind observations [8]. Several studies have demonstrated that the ERA5 surface wind data has the highest consistency with field observations compared to JRA55, CFSR/NCEP, and ERA-Interim Reanalysis 1 (R1) [18]. Compared with the Advanced Scattered Advanced Wind Scattering (ASCAT) ocean vector wind observation, ERA5 performs better than ERA Interim in terms of instantaneous root mean square wind speed consistency, average value, and transient wind error [19].

Although reanalysis data can cover global regions, the accuracy of local data in some regions is still insufficient deficient due to deficient observational data. Evaluating reanalysis data is a crucial process in improving the accuracy of reanalysis data, subsequently, enhancing confidence in its application. Several performance assessments of ERA5 wind reanalysis data were conducted using observed from various locations, including Zhejiang nearshore waters [20], the South China Sea [21], North America [22], Europe [23], and global regions [24,25]. The accuracy assessment of the ERA5 wind reanalysis data over China’s coastal water is quite limited owing to the lack of observational data [26].

The objective of present work is twofold. First of all, a systematic comparison was made between the ERA5 reanalysis data and the in-situ measurements from 19 wind towers offshore China, examining the potential application of this wind data. We will verify the consistency between ERA5 wind field reanalysis and available observed winds overall the coastal regions of China through several statistical analysis tools. If the evaluation proposed in the study demonstrates the capability of ERA5 reanalysis to describe wind fields near the coastal of China, further studies on energy resources analysis and climate model validation can be considered in the future. The complete distribution and high spatial-temporal resolution of ERA5, as well as consistency with other atmospheric variables, will help to the understanding of wind variability and its mechanisms in advance. The second objective is to describe the wind distribution and behavior offshore China Sea by inspecting several statistical calculations and using the wind speed frequency distribution.

The structure of the paper is arranged as follows. In Section 2, ERA5 reanalysis wind data, wind measurement from 19 wind towers are described. The method for evaluation and comparison adopted in this study are briefly introduced in Section 3. Section 4 presents the results in detail, and a comprehensive discussion is given. Section 5 concludes with a summary of the main findings.

## 2. Data

### 2.1. ERA5 data

ERA5 is the fifth-generation reanalysis produced by the ECMWF, providing a range of atmospheric, land surface, and sea state variables covering the globe [24,27]. ERA5 combines the model data with worldwide observations, utilizing 4D-Var data assimilation and model forecasts in Cycle 41R2 of the ECMWF Integrated Forecast System (IFS), to generate a consistent and complete global dataset. The temporal range of the ERA5 reanalysis data is from 1979 to the present, providing hourly estimates for numerous atmospheric, ocean wave, and land surface variables. The atmospheric components are interpolated to 37 pressure levels from the surface up to 1 Pa. In this study, only wind data at 10m and 100 m are used, keeping consistent with the observation data. The horizontal grid resolution of the ERA5 reanalysis data was 0.25° × 0.25°, equivalent to approximately 31 km. ERA5 is freely accessible via the Copernicus Climate Change Service (C3S, https://climate.copernicus.eu/) funded by the European Union. The validation process of ERA5 is ongoing, involving comparison with previous observations and reanalysis at regional or local scales, primarily in Europe [18,23]. Further more details information on ERA5 characteristics, please refer to [24].

In this work, the horizontal wind field components, eastward component (u), and northward component (v) at heights of 10 m and 100 m are used to calculate wind speed as $\sqrt{u^{2}+v^{2}}$ at the cells where observational data is located. The method of selection data from ERA5 follows Molina et al. [28], which extracts the cell wind variation at the specific points (latitude and longitude) in which the observation stations are located. Time range with no observational data are not used.

### 2.2. Observation data

**Fig 1:**
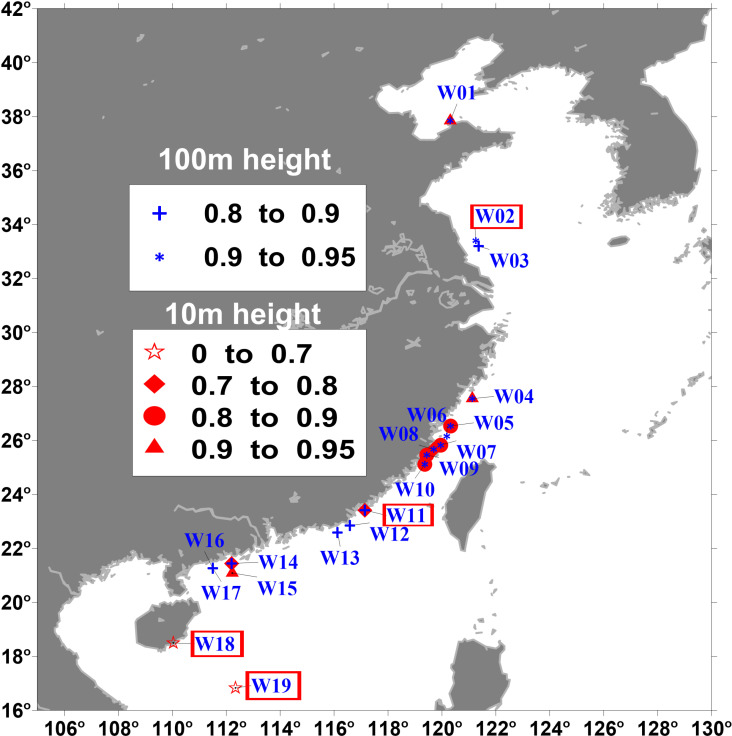
Location on the map of each wind observation station. The color scale represents the Perkins Skill Score for each location. The wind frequency distribution of the stations marked with a red square symbol is shown in Fig 5.

The wind observation data was obtained by wind measurement devices installed on wind towers deployed in the China’s offshore areas. The wind monitors (Type 05106) are manufactured by R. M. Young Company, with the wind speed accuracy of ± 0.3 m/s. Wind data is recorded every 10 minute at heights of 10 m and 100 m above the mean sea surface, respectively. Quality inspection and control procedures is used to eliminate bad data before use, such as singular values, default values, and neighbor. The detailed locations of the 19 observation stations are shown in Fig 1, and more information about the data can be found in Table 1.

**Table 1:** Detailed information of the observation datasets.

ID	Station Name	Location		Period (day/month/year)
		Longitude (E)	Latitude (N)	
W01	ShanDong peninsula	120.31°	37.85°	1/1/2022-31/12/2022
W02	DaFeng H11	121.26°	33.40°	1/1/2015-31/12/2015
W03	DaFeng H8	121.36°	33.20°	1/1/2022-31/12/2022
W04	WenZhou RuiAn	121.13°	27.55°	1/1/2022-31/12/2022
W05	NingDe XiaPu B	120.32°	26.54°	1/12/2019-31/12/2020
W06	LianJiang offshore	120.18°	26.16°	1/5/2022-30/4/2023
W07	PingTan offshore	119.95°	25.83°	1/8/2019-31/7/2020
W08	PingTan DaLian Island	119.69°	25.67°	1/7/2015-30/6/2016
W09	XinHua Bay	119.43°	25.46°	1/1/2015-31/12/2016
W10	PingHai Bay	119.37°	25.12°	1/5/2012-30/4/2014
W11	SanTou NanAo	117.14°	23.42°	1/4/2010-31/3/2012
W12	JieYang JingHai	116.58°	22.85°	1/9/2010-1/9/2011
W13	SanWei Jiazi	116.13°	22.59°	1/9/2019-31/8/2020
W14	YangJiang Nan Peng	112.20°	21.44°	1/1/2016-31/12/2016
W15	YangJiang FanShi	112.22°	21.09°	1/4/2020-31/3/2021
W16	YangJiang ShaPa Ⅰ	111.50°	21.26°	1/1/2018-31/12/2018
W17	YangJiang ShaPa Ⅱ	111.50°	21.26°	1/1/2018-31/12/2018
W18	LingShui	110.03°	18.50°	1/1/2015-31/12/2015
W19	Yong Xing	112.34°	16.83°	1/1/2015-31/12/2015

## 3. Method

### 3.1. Statistical evaluation

In order to judge the quality of the ERA5 wind data in the China Sea, the observation data by wind tower are regarded as the “true value”. The mean absolute error (MAE), bias (*B*), and coefficient of determination (*R*^*2*^) are firstly calculated. They are defined as follows:


$$MAE=\frac{1}{N}\sum_{i=1}^{N}\left|r_{i}-o_{i}\right|$$ (1)

$$B=\frac{1}{N}\sum_{i=1}^{N}\left(r_{i}-o_{i}\right)$$ (2)

$$R^{2}=1-\frac{\sum_{i=1}^{N}{\left(r_{i}-o_{i}\right)}^{2}}{\sum_{i=1}^{N}{\left(o_{i}-\bar{o_{i}}\right)}^{2}}$$ (3)

being *r* reanalysis values, *o* observational data, and *N* the number of observations. Taylor diagram is used to visualize the statistical summary of how well pattern match each other in terms of their standard deviation (*σ*), correlation coefficient *R*, CRMSE, and the between reanalysis and observations in a single chart [29]. The three statistic parameters are calculated is as follows:


$$\sigma =\sqrt{\frac{\sum_{i=1}^{n}{\left(x_{i}-\mu \right)}^{2}}{N}}$$ (4)

$$R=\frac{\frac{1}{N}\sum_{i=1}^{n}\left(r_{i}-\bar{r_{i}}\right)\left(o_{i}-\bar{o_{i}}\right)}{\sigma_{r}\sigma_{o}}$$ (5)

$$CRMSE=\sqrt{\frac{\sum_{i=1}^{n}{\left(\left(r_{i}-\bar{r_{i}}\right)-\left(O_{i}-\bar{o_{i}}\right)\right)}^{2}}{N}}$$ (6)

where *x* the wind speed sample and *μ* the mean of the wind speed. Previous studies shown that reanalysis data provides a poor representation of hourly variability in wind speed at individual locations, compared with observed wind speed, whereas 6 hours or longer interval data show better results [30]. Here, we evaluated the hourly, 6- and 24-hourly time frequencies to study this behavior. During the observation period of each measurement station, the centered root-mean-square error (CRMSE), and Pearson’s correlation coefficient, and standard deviation (SD) between ERA5 and observation data are calculated on three temporal scales, to analyze the consistency between ERA5 and in-situ observations.

In the normalized diagram, the CRMSE and the two standard deviations of each time series are normalized by the standard deviation of the corresponding observed field. The azimuthal position represents the Pearson correlation coefficient, zero and 90 degrees indicate a correlation of 1 and 0, respectively. The standard deviation ratio between the reanalysis and observations is represented by the radial distance from the origin. The CRMSE of the reanalysis is depicted as the radial distance from the position of the observation, and the observation is located at *s* = 1, R = 1, and CRMSE = 0 in the normalized Taylor diagram.

### 3.2. Wind parameters

For each station, we calculated several specific wind parameters spanning the entire period, incorporating scores and a matrix of relevant parameters to comprehensively describe wind characteristics, and the objective is to investigate the combined relations among these parameters. The proposed wind parameters are the following:

A) The coefficient of variation (CV) serves as a dimensionless measure of wind variability. It is calculated using the standard deviation of wind normalized with the mean wind, enabling comparisons across different locations. The mathematical formula for CV is as follows:

$$CV=\frac{s}{\mu }$$ (7)

B) Cut-in and cut-out threshold rates. Cut-in and cut-out rates are the thresholds for the operation of wind turbines. Cut-in is the wind speed at which the turbine starts generating electricity, and cut-out is the wind speed at which production needs to be shut down to prevent damage on wind turbines [31]. These values depend on the wind specific turbine. Taking turbine model GW154/6700 manufactured by Goldwind Science and Technology as example, the technical specification is given in Table 2, and the Cut-in and Cut-out wind speed are 3 and 25 m/s, respectively, which are considered as commonly values for standard wind turbines. In this way, correlation analysis is conducted using the percentage of hours at each observation station that are below (above) these fixed thresholds.

**Table 2:** General technical specification for GW154/6700.

Component	Unit	Specification
Rated power	kW	6700
Wind class	/	IEC S
Power adjustment method	/	Variable pitch variable-speed control
Rotor diameter	M	154
Hub height	m	S
Cut-in wind speed	m/s	3
Rated wind speed(static)	m/s	12.1
Cut-out wind speed (10mins average)	m/s	25
Extreme wind speed (3s average)	m/s	77.84
Iref	/	0.14
Operating temperature	°C	-20 to + 40
WTGS survival temperature	°C	-30 to + 50
Design life time	Year	≥25

### 3.3. Data evaluation: Score

To measure the degree of closeness between the sample distribution function and the probabilistic distribution function, the Kolmogorov–Smirnov (K-S) test used to determine if two samples come from populations with the same distribution. This technique utilizes F(x) and S(x) to denote the estimated cumulative probability of ERA5 and observational sample, respectively. The test’s statistic, D, is derived as the maximum absolute difference between F(x) and S(x), i.e.,


$$D=max\;\left|F\left(x\right)-S\left(x\right)\right|$$ (8)

Additionally, the critical value that obeys the probabilistic distribution (P) based on the sample size and significance level (significance level was set at 0.01) is provided. If D is less than or equal to P, it indicates that two sample follows the same probabilistic distribution. In this study, passing this test indicates that there is no statistically significant difference between the PDFs of ERA5 and observational wind speed distributions.

It should be mentioned that other statistical tests exist which examines similar parts of the model-observed statistical space. However, it is not clear how to sum across these PDF-based statistics. So, Perkins et al. developed an alternative metric that appears to be a very simple but very useful measure of similarity between two PDFs, which allows a comparison across the entire PDF [32]. The method is successfully used to evaluate temperature and precipitation results from climate models against available observations [32]. This approach also has been used in wind speed studies to compare modeling results with observations [33]. The method is based on the amount of overlap between probability density functions (PDFs) of the wind speed for observations (the reference data) and ERA5, looking for their grade of matching between model output and to the reference data. Two skill scores were used to compare the PDFs: Perkins Skill Score [32] and Brier Score [34].


$$\text{Perkins Skill Score=}\sum_{\text{i=1}}^{\text{n}}\text{minimum}\;\left({\text{p}}_{\text{mi}}\text{, }{\text{p}}_{\text{oi}}\right)$$ (9)

$$\text{Brier Score}=\frac{\text{1}}{\text{n}}\sum_{\text{i=1}}^{\text{n}}{\left({\text{p}}_{\text{mi}}-{\text{p}}_{\text{oi}}\right)}^{\text{2}}$$ (10)

In which the ${\text{p}}_{\text{mi}}$ and ${\text{p}}_{\text{oi}}$ are the probability values in the *i*th bin (here, 0.5 m/s has been used as bin size) from total n bins obtaining the PDFs of observation and model data, respectively. The Perkins Skill Score evaluates the similarity between two PDFs by calculating the accumulative minimum probability of the modeled and observed distribution for each bin. Both PDF areas will coincide, i.e., the score equals to 1, if ERA5 compares perfectly observed values. Any other situation would result in a value between 0 and 1. And Brier Score calculates the mean square root error for probability simulations. Thus, a smaller Brier Score can indicate the better performance of the reanalysis data. It is noted that this method of comparison that the PDF distribution of the reanalysis represents an area of 31 km, while the frequency distribution of the observations refers to a specific location. Thus, a misrepresentation of extremes could be obtained even with good scores [32]. Significant differences would not be reflected on the score due to the quite small area around tails.

## 4. Results and discussion

### 4.1. Statistical comparison of ERA5 against observations

**Fig 2:**
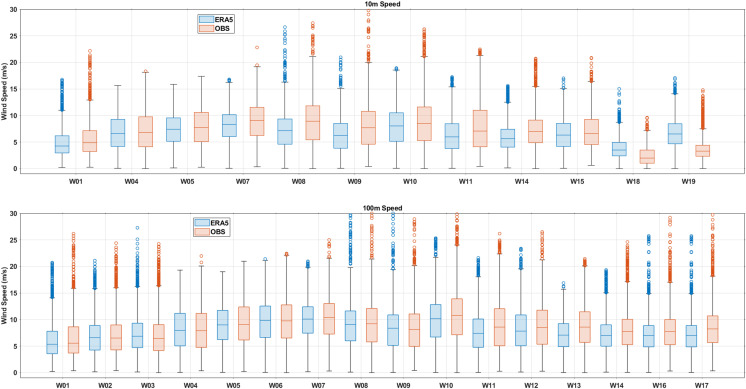
Wind speed box plots at observation stations (red) and ERA5 cells (green) at heights of 10m and 100m, respectively. The boundary of the boxes represents the positions of the 25th and 75th percentiles, and the middle line represents the 50th percentile. The upper whisker is a maximum value, while the lower whisker is a minimum value, and cycle indicates the outliers exceeding three times the standard deviation.

As shown in Fig 2, the spatial variability of wind is comprehensively evaluated through a box plot of the 19 locations (observation stations and corresponding reanalysis grid cells) near the coast of China. Each box represents the hourly data distribution of a specific station during the observation period. Overall, the basic statistical characteristic between ERA5 reanalysis and observation of wind speed shows good consistency. Especially, the minimum wind speed at heights of 10 m and 100 m is consistent. However, the interquartile range (75th percentile and 25^th^ percentile) reflecting the degree of data dispersion, exhibits some differences at all stations, especially W09 and W11 stations, observed hourly wind speed shows a wider range at the height of 10 m. Each box exhibits an approximate symmetry for reanalysis and observation, where the lower percentile width (25–50) is approximately equal to higher percentile width (50–75). The median wind speed between ERA5 and observation is also almost equal, with the median wind speed at most stations being 5-10 m/s at the height of 10 m, and slightly larger at the height of 100 m.

The data points (indicated by cycles in Fig 2) located outside the upper and lower edge areas in the box diagram represent outliers in the data, usually caused by extreme weather events such as typhoons. The upper extreme wind values in observed data are usually larger than ERA5, which means that the observed wind inspects larger extreme wind speeds. This is especially true at the height of 10 m, where discrepancies in extreme values between reanalysis and observations are more pronounce. This means ERA5 seems to more significantly underestimate extreme winds near the sea surface. These comparisons indicate the ERA5 typically underestimates the wind speed, especially in the high-grade winds, which is in consistent with previous analyses results for global or some region studies [23,34–36]. Here, the average values for all the 19 locations in China’s coastal waters, an overall consistency has been found. However, ERA5 shows discrepancies in its ability to represent extreme wind speed, due to the strong small scale characteristics of wind, especially occurs in extreme weather conditions.

Tables 3 and 4 give the statistical results from the comparison of wind speeds from ERA5 at different observation sites and heights against those measured by the wind tower. The MAE is around 2 m/s for all stations, the most of stations exhibit the negative bias, and their R^2^ values is around 0.7. The negative bias implies ERA5 underestimates the actual wind speed. Two skill scores (Perkins Skill Score and Brier Score) comparing the PDFs similarity between ERA5 and measured wind speed are given in Tables 3 and 4. It exhibits higher skill score (larger Perkins Skill Score and lower Brier Score) at the height of 100m. The worst skill scores are present at W18 and W19 stations. The result of the K–S test is given in the last column of Tables 3 and 4. In the present study, the null hypothesis for K-S test was that ERA5 wind speed data fit the observational wind distribution. ’Pass’ means that the K–S test did not reject the null hypothesis, and ’Fail’ indicates that the K–S test rejected the null hypothesis. As is shown, none of ERA5 wind speed data with the higher skill score could pass all the K–S tests at the 1% significance level. In addition, the stations with lower skill scores generally failed the tests.

**Table 3:** Wind speed statistical evaluation between observation stations and ERA5 data at heights of 10m.

Station	MAR (m/s)	B (m/s)	R^2^	Perkins Skill Score	Brier Score (×10^-5^)	KS Test
W01	2.29	-0.73	0.73	0.90	17.1	Pass
W04	1.93	-0.23	0.78	0.91	3.68	Fail
W05	1.97	-0.57	0.75	0.88	8.95	Pass
W07	2.07	-0.82	0.72	0.86	7.40	Pass
W08	2.59	-1.62	0.69	0.76	12	Pass
W09	2.42	-1.54	0.73	0.82	8.01	Pass
W10	2.10	-0.75	0.71	0.89	3.01	Pass
W11	2.55	-1.41	0.55	0.75	19.7	Fail
W14	2.18	-1.25	0.84	0.80	17.7	Pass
W15	1.74	-0.49	0.88	0.90	3.54	Pass
W18	1.91	1.41	0.47	0.66	85.0	Fail
W19	3.25	3.18	0.67	0.45	78.8	Fail

**Table 4:** Wind speed statistical evaluation between observation stations and ERA5 data at heights of 100m.

Station	MAR (m/s)	B (m/s)	R^2^	Perkins Skill Score	Brier Score (×10^-5^)	KS Test
W01	2.71	-0.47	0.79	0.94	4.64	Pass
W02	2.20	-0.22	0.83	0.95	1.01	Pass
W03	3.91	0.21	0.85	0.90	4.19	Fail
W04	2.29	0.09	0.91	0.94	1.49	Fail
W05	2.28	-0.36	0.81	0.90	3.76	Pass
W06	2.20	-0.07	0.88	0.94	1.45	Pass
W07	2.24	-0.38	0.81	0.91	2.23	Pass
W08	2.13	-0.13	0.86	0.93	1.00	Pass
W09	2.09	0.003	0.91	0.94	0.89	Pass
W10	2.47	-0.87	0.81	0.90	1.90	Pass
W11	2.71	-1.19	0.64	0.82	8.35	Fail
W12	2.15	-0.69	0.77	0.89	3.45	Pass
W13	2.51	-1.59	0.74	0.81	12.7	Pass
W14	2.23	-0.68	0.81	0.89	4.82	Pass
W16	2.11	-0.75	0.81	0.88	3.93	Pass
W17	2.40	-1.30	0.79	0.82	8.12	Pass

**Fig 3:**
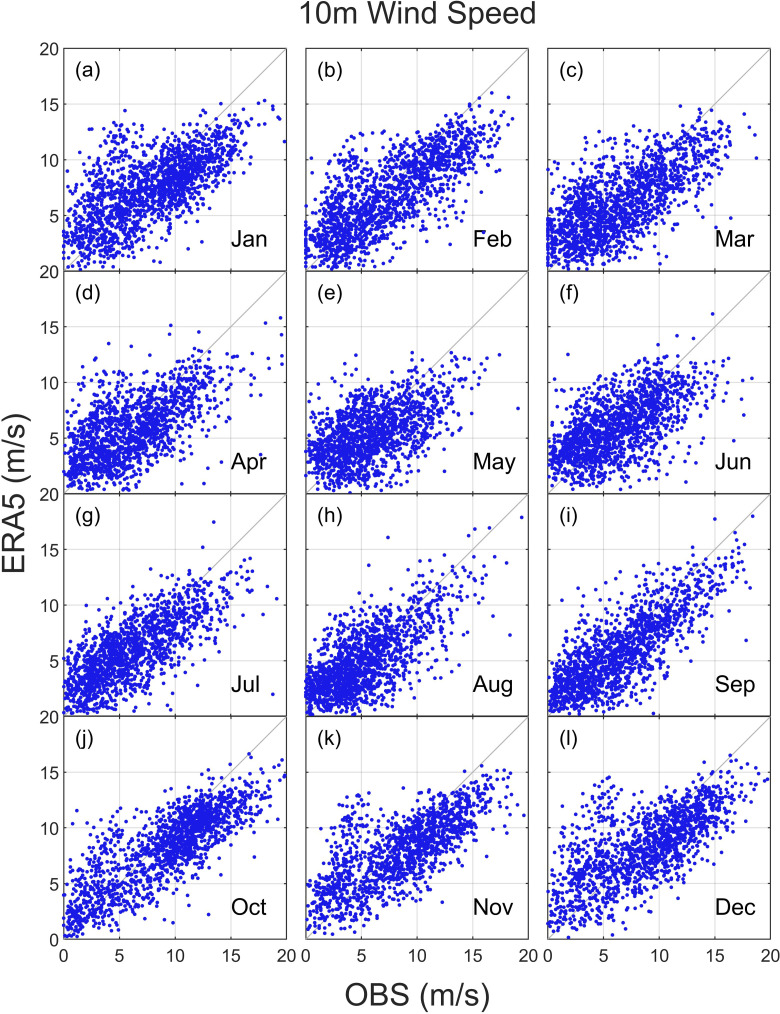
Comparison hourly means of wind speeds from 19 wind observation stations with ERA5 reanalysis wind data in the China’s offshore water. The diagonal line indicates a perfect fit.

To investigate the monthly discrepancies for all observation locations, Fig 3 presents a scatter plot of wind speed in the different month. Dots represent hourly wind speed pairs for each ERA5-cells/station. Due to the majority of points being close to the diagonal, a close overall correspondence was obtained between the two datasets. This relation is stronger from May to Oct than from November to March, in which high wind speed (more than 5 m/s) and larger biases are present, although the relative errors are similar in all months. For larger wind speed, it is worth noting that reanalysis is difficult to accurately represent these high wind speeds, because they are not results of specific location as described in the observational data, but rather results of larger area (a grid cell of several km), thus allowing some smoothing of wind variability in the region [18].

**Fig 4:**
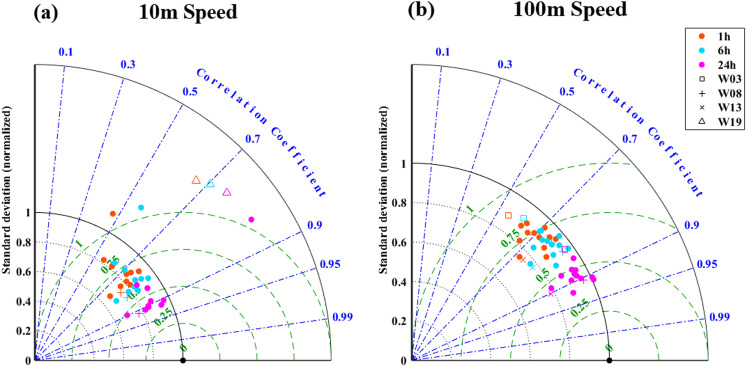
The normalized Taylor plots of wind observations and ERA5 reanalysis compared the hourly (red dots), 6-hour (green dots), and 24-hour (pink dots) data of each station during the measurement period. The Pearson correlation coefficient between ERA5 and observation is given by the azimuthal position, and the standard deviation ratio between the ERA5 and the observed values is represented by the radial distance from the origin. The reanalyzed CRMSE is represented as the radial distance from the position of the observation (green semicircle). In this case, when the point is closer to the observation σ = 1, R = 1, and CRMSE = 0, reanalysis will represent better observed data. Stations marked with squares and symbols represent the stations with the highest scores, while stations marked with crosses and triangles represent the stations with the lowest scores.

To evaluate the ability of ERA5 to reproduce the temporal wind variability at different time scales, Fig 4 compares ERA5 reanalysis with direct wind measurements at hourly, 6-hour, and 24-hour averaged intervals, utilizing a Taylor diagram to assess CRMSE, Pearson correlation, and standard deviation (SD). As the CRMSE approaches 0, the SD and correlation approach 1, it indicates that the ERA5 wind speed time series is better aligned with observational data. When comparing hourly statistics with daily statistics, the results highlight significant differences. At a height of 10 m (Fig 4a), the hourly data exhibits a CRMSE higher than 0.5 in all data points, with a correlation coefficient between 0.52 and 0.73 for most of the stations, only a few points having correlation values as low as 0.48. whereas the 24-hour data show a higher correlation coefficient, with most stations having correlation coefficients between 0.8 and 0.9, accompanied by a lower CRMSE ( < 0.5), and a standard deviation closer to the observations compared to hourly results. For an average of 6-hour, the correlation of most data points is about 0.85, which is also better than the hourly scales correlation, with similar standard deviations and lower CRMSE values ( ≈ 0.45). It should be pointed out that several stations show high CRMSE, which was greater than 1. These results are consistent with previous studies [23,31,37,38], in which wind speeds from different reanalysis products were compared with meteorological data or flux tower observations from different regions around the world [38].

At a height of 100 m, Taylor diagrams is also shown in Fig 4b. The analysis reflects that ERA5 and observations winds are similar in terms of correlation, CRMSE, and SD to the situation observed at a height of 10m in Fig 4a. In addition, there is better consistent between ERA5 and the entire observed values. If average deviation or RMSE is calculated, the absolute biases range is about ± 2 m/s, which is related to time frequency and better and worse representations of the station, that is consistent with the normalized Taylor diagrams shown in Fig 4.

### 4.2. ERA5 evaluation: score

In the previous section, statistical evaluation was conducted, using the CRMSE and correlation coefficients, to assist in analyzing temporal variability and bias. Although the correlation assessment evaluates the temporal evolution of wind speed, the station-specific scores allow for a comprehensive analysis of ERA5, which is consistent with observations of the entire frequency distribution of wind values [32], without considering the temporal aspect. Therefore, for some stations, a preference correlation may coexist with large deviations throughout the entire time series, resulting in lower scores. Therefore, a comprehensive comparison must be made from different perspectives.

Fig 1 shows the first overview of the scores calculated based on hourly data from 19 locations, represented by classical symbols according to calculated Perkins Skill Scores. In terms of quantitative indicators, the most of Perkins Skill Scores at the height of 100m are close to or greater than 90%, and Perkins Skill Score for five stations is between 0.8 and 0.9. Their Brier Scores are also negligible, indicating that ERA5 has a good ability to reproduce the observed frequency distribution. At the height of 10m, the Perkins Skill Score show wide range (0.45 ~ 0.95). It is easy understand that wind simulation for the air-sea boundary layer is more difficult due to complex processes of air-sea interaction. Scores also show significant geographical dependence, two locations (W18 and W19) with the lowest scores below 0.7 are located near Hainan Island. The region with high scores is mainly located the north of the Taiwan Strait. Stations with higher scores tend to correspond to lower CRMSE and stand derivation values, as well as higher correlation coefficients, as shown in the Taylor diagram (Fig 4). Inspecting mean deviation, as smaller average biases are usually associated with smaller scores. When the score is around 0.6, the absolute deviation may be as high as 2 m/s, and when the score is 0.9 or higher, the absolute deviation is less than 1 m/s.

The simulation of the change of wind speed during the typhoon was not accurate by ERA5, and there was a significant overestimation of low wind speed and underestimation of high speed during the typhoon periods [18–20]. While South China Sea is the most severely affected by typhoons, so ERA5 shows the higher simulation skill in the northern region than the southern. Next, W18 and W19 station near the Hainan Island show the worst simulation skill according to various statistic evaluation. Except for the influence of typhoon, the complex topography and sea–land boundaries are assumed as important factors, and needs to be further studied and strengthened in the future.

In addition, compared to hourly data shown in Fig 4, we observed larger scores for 6-hourly and 24-hourly data (Figure omitted). These improved scores are consistent with the findings in the Taylor diagrams analysis (Fig 4). When evaluated using scoring metrics, ERA5 shows superior performance compared to in-situ measurement data, particularly for 6- and 24-hourly averaged data. The reason behind this is that when obtaining 24-hour data, small-scale (hourly) differences are smoothed out. As previously mentioned earlier, the smoothing effect of time averaging enhances the ERA5 model’s ability to represent highly variable conditions. Previous studies mainly relied on 24-hour analysis, so any similarities or differences found in our study can strengthen or supplement these previous studies. Given that both ERA5 and in-situ measurement both use hourly data as their reference temporal resolution, comparative analysis of different time frequencies may enrich previous studies with lower spatial-temporal resolutions, providing new insight into wind characteristics.

**Fig 5:**
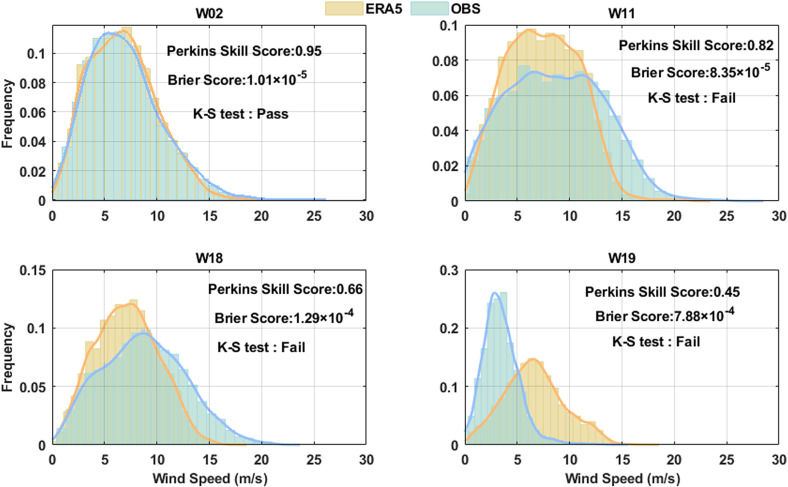
Frequency distribution of the hourly ERA5 reanalysis vs observations illustrating the best score (a: W02 station and b: W11 station) and the poorest score (c: W18 station and b: W19 station). The location of each station can be seen marked with station ID in Fig 1.

To inspect some specific cases where there is slight difference between wind distributions, what is more interesting is that these differences are significant. Fig 5 gives a comparison of the probability density function (PDF) of the wind speeds between the ERA5 and observations at the several selected stations, where the one with highest Perkins Skill Score (a: W03 station), one with moderate Perkins Skill Score (b: W11 station) and two worst (c: W18 station, d: W19 station) stations were selected. For the best one, the distributions of observation and reanalysis almost perfectly matches with Perkins Skill Score reaching 0.95, and pass the K-S test. For W11 station, with smaller Perkins Skill Score (0.82), the ERA5 underestimates higher wind speed (in the range of 12-20 m/s) and overestimates lower wind speed frequencies (in the range of 2-12 m/s), and K-S test fail. For two worst station (Fig 5c and 5d), K-S test fail. At W18 station, ERA5 exhibits higher frequency in the low wind speed range (2-10 m/s). Conversely, for W19 station, with the smallest Perkins Skill Score (only 0.45), the ERA5 significantly overestimates high wind speed and underestimates low wind speed frequencies. Overestimation of gentle winds and underestimation of strong winds in reanalysis data is a common and known feature, as demonstrated in previous studies [30]. In previous works, the underestimation of strong wind frequency and overestimation of gentle wind frequency were also studied for model analysis [39]. This underestimation of high wind speed in model analysis is related to the numerical solution of the dynamic equations to each point, depending on the defined grid size, which may lead to inaccurate descriptions and a certain degree of smoothness. The higher the resolution of model, the smaller this problem is expected to be. In addition, parameterizations in models also can lead to an underestimation of the most extreme events [30]. Overall, the ERA5 provides very similar PDF on all sites near the offshore water of China. The discrepancies between corresponding bars are not exceed 5%. However, the ERA5 overestimates the low range of the wind speeds (approximately 0 m/s to 10 m/s) and underestimates high-wind-speed events (the right tail of the PDF) in certain regions. The ERA5 Strong wind deviations compared with observations are almost not reflected in scores, as tail frequencies have little effect on the final number [32].

**Fig 6:**
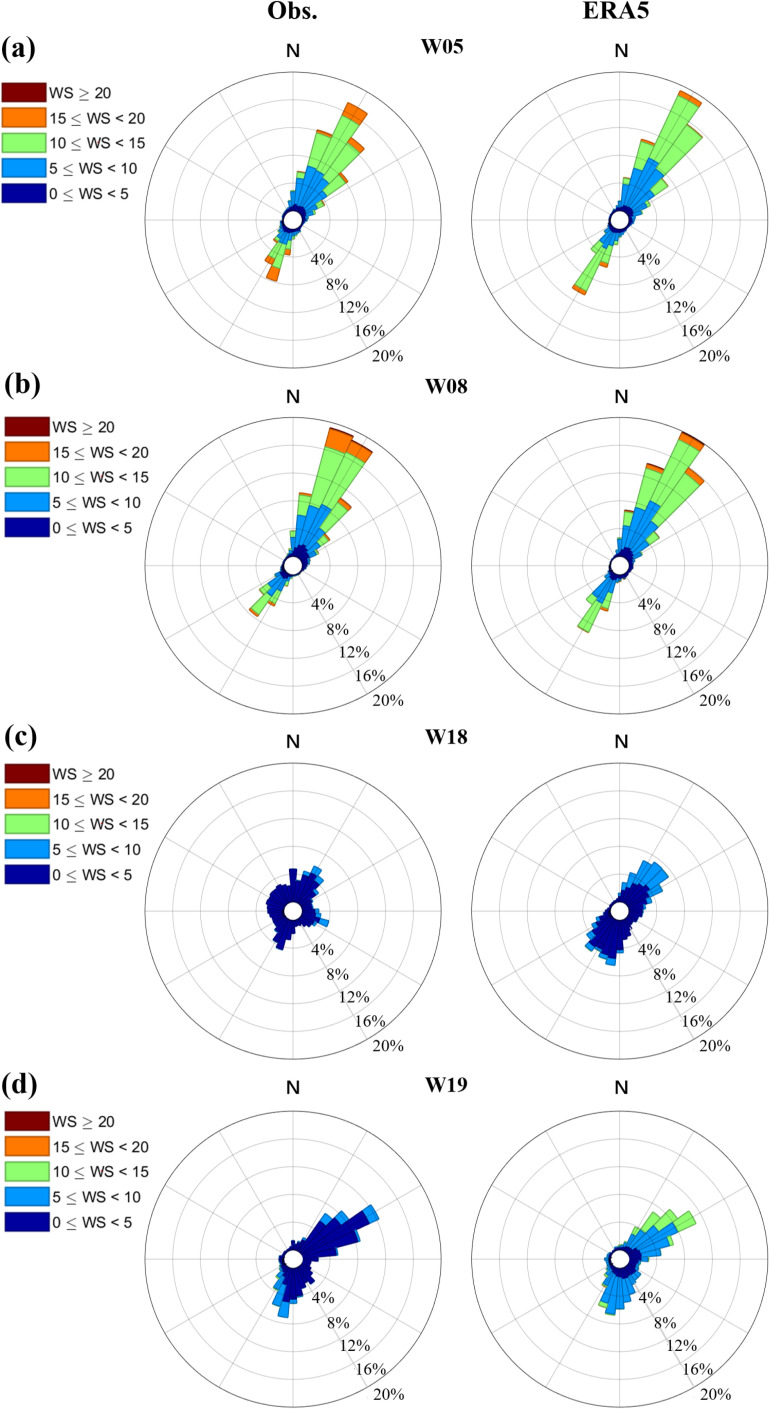
Comparison of wind roses between ERA5 reanalysis and in-situ measured data. a: W03 station, b: W08 station, c: W13 station and d: W19 station. The location of station is marked in Fig 1 with station ID.

The wind rose diagrams were drawn for four sites based on in-situ data and ERA5 data, and shown in Fig 6. The results indicate that ERA5 data can well describe the general characteristics of coastal winds in China. At the highest scoring stations W02 and W11, the wind directions and speed of ERA5 data are almost identical to the observational data. However, for W18 and W19 stations with the lowest scores, the wind direction deviation is relatively significant. The frequency of wind speed of 5-10 m/s at W18 station is overestimated, and the true wind frequency is higher in the northwest and southeast directions, whilst in ERA5, they mainly come from the southwest and northeast directions. At W19 station, wind speed of 10-15 m/s are overestimated in frequency, but the observed and ERA5 wind directions are usually consistent.

### 4.3. Wind parameters analysis

**Fig 7:**
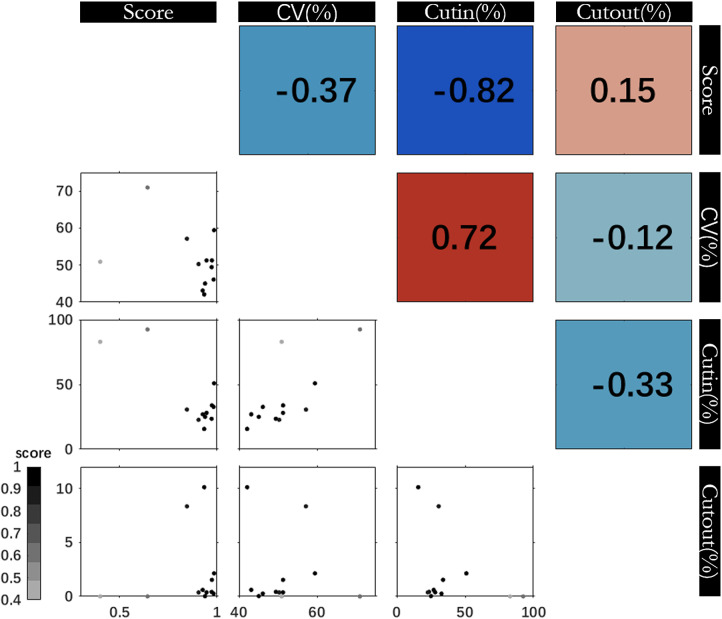
Scatter plot matrix of wind parameters for hourly wind speed observations data at each location: score, CV (coefficient of variation), frequency of values above the Cut-out (25 m/s) and under the Cut-in (3 m/s) rates. Upper panels show the Pearson’s correlation for each variable pairs and lower panels show scatter plots. Grayscale indicates ranges of score from lower to higher scores.

Wind characteristics are analyzed by combining different wind parameters. Fig 7 shows scatter plots of several wind parameter pairs for 19 observation stations. The lower triangle in Fig 7 presents the matrix of scatter plot of parameter pairs between CV and percentage of data below/above the cut-in and cut-out threshold. Scores calculated between measurements and ERA5 are also displayed in grayscale. The upper panel triangle in Fig 7 provides Pearson correlation coefficients for measuring these scatter plots. If scatter plots show a point cloud close to a straight line, the Pearson correlation coefficient will be close to 1 (red color) or -1 (blue color). The more scattered the points, the lower the correlation coefficient.

Scatter plot matrix of wind parameters is used for the overall analysis of wind statistics combinations. The results show that CV is significantly correlated with Cut-in, with a Pearson correlation coefficient of 0.72, but CV is slightly correlated with Cut-out, with a correlation coefficient of only -0.12. This means that winds with larger CV usually accompany with more frequent breezes, i.e., more variable wind conditions, while smaller breezes are associated with less variable and more stable wind values. In addition, there is no high correlation between the Cut-in frequency (10-75% of hours below 3m/s) and Cut-out frequency (1 to 13% of the hours above 25 m/s), with a correlation coefficient of only -0.33, indicating that the smaller amount of strong wind date is not related to a larger light wind amount.

To examine the impact of data time interval on the analysis parameters, we also compare the hourly data with 6- and 24-hourly time-scales (Figure omitted). The results indicate that as time average increases, the frequency below the Cut-in increase, while the Cur-out frequencies decreases. The percentage of hourly data above the threshold of 25 m/s is greater than daily averaged data. And in the case of cut-in frequencies, the opposite situation occurs. It should be pointed out that there is no linear change in magnitude from hourly data to daily averages. Cannon et al. also pointed out these discrepancies by comparing wind speed at the 6-hourly with hourly data [30].

In order to investigate the relevance of wind parameters obtained, the first row of Fig 7 shows scores correlation with other parameters calculated based on hourly data. The score is weakly negatively correlation with the CV (-0.37) and strongly negatively correlation with Cut-in rate (-0.82). This negative correlation indicates that stations with lower breeze frequency or smaller CV are associated with better ERA5 performance. It sounds reasonable, ERA5 present the more frequent breeze due to resolution limitations. However, as shown by larger expansion points, there is no significant linear relationship between score and Cut-out frequency The score and CV present a more significant correlation with frequency of data above the Cut-in (-0.82 and 0.72, respectively).

## 5. Conclusions

The ERA5 reanalysis wind data were evaluated and analyzed based on 19 in-situ observations of wind speed in China's coastal waters. To our knowledge, this is the largest in-situ measurement data covering the entire the coastal water of China. Furthermore, the long time series and hourly data are available, which were difficult to find in previous studies. This is crucial for a better understanding of wind characteristics, and the hourly time interval are more interesting when combined with the newest ERA5 reanalysis data.

In-situ wind observations exhibit significant temporal variation, with clear seasonal cycles of northeast winds in winter and southwest winds in summer. ERA5 reanalysis data accurately reproduced this temporal pattern. The consistency between ERA5 and in-situ observation is better in the daily average data. The time correlation between ERA5 reanalysis and observations of daily data is high, with values ranging from 0.6 to 0.9 for many stations. When average interval of the data changes from 6-hourly and to hourly, the high correlations with small CRMSE and standard deviations are further decreases. Several wind parameters were also investigated for each observation station, allowing for a detailed analysis of the wind characteristics in China’s offshore waters.

For each station, the various statistical methods are utilized for the overall evaluation. Especially, calculate wind speed frequency distributions, and use score to evaluate ERA5 performance. The score of most stations exceeds 0.8, which means that ERA5 reanalysis wind data reproduces the frequency distribution of wind speed observed near the coast of China. The scores do exhibit a clear geographical dependence, with many stations in the BoHai Sea, Yellow Sea and East China Sea having higher scores. The worst score is 0.45, appearing at two specific stations near the Hainan Island. ERA5 shows the higher simulation skill in the northern region than the southern due to the influence of high-frequency typhoon in the South China Sea. The complex topography and sea–land boundaries are assumed as important factors which lead to the worst simulation skill in the W18 and W19 stations. The scores of 6-hourly and 24-hourly data are higher than those of hourly data, which is consistent with the correlation shown in the Taylor diagram. This result is expected, as the smoothing effect bring better performance compared to observations results.

To sum up, both on an hourly or daily time scale, several statistics for comparison between ERA5 and in-situ measurements are reasonably good. Therefore, ERA5 reanalysis data is allowed to be used to validate climate models with dynamical spatial-temporal consistency. Especially when observational data are not available or the time series is not enough long, ERA5 reanalysis wind data is very useful. However, it is worth noting that the ERA5 reanalysis underestimates the extreme wind, especially under the extreme weather conditions, Future research should consider higher resolution wind product, strengthen extreme wind simulation, because it is important to examine extreme wind events for wind farm design.
